# Point of view filming and the elicitation interview

**DOI:** 10.1007/s40037-016-0278-0

**Published:** 2016-07-20

**Authors:** Jonathan Skinner, Gerard J Gormley

**Affiliations:** 1 Department of Life Sciences, University of Roehampton, London, UK; 2 School of Medicine, Dentistry and Biomedical Sciences, Centre for Medical Education, Queen’s University Belfast, Belfast, Northern Ireland

**Keywords:** Point of View, Elicitation interview, Simulation, Pedagogy

## Abstract

Face-to-face interviews are a fundamental research tool in qualitative research. Whilst this form of data collection can provide many valuable insights, it can often fall short of providing a complete picture of a research subject’s experiences. Point of view (PoV) interviewing is an elicitation technique used in the social sciences as a means of enriching data obtained from research interviews. Recording research subjects’ first person perspectives, for example by wearing digital video glasses, can afford deeper insights into their experiences. PoV interviewing can promote making *visible* the *unverbalizable* and does not rely as much on memory as the traditional interview. The use of such relatively inexpensive technology is gaining interest in health profession educational research and pedagogy, such as dynamic simulation-based learning and research activities. In this interview, Dr Gerry Gormley (a medical education researcher) talks to Dr Jonathan Skinner (an anthropologist with an interest in PoV interviewing), exploring some of the many crossover implications with PoV interviewing for medical education research and practice.

## Introduction

**Fig. 1:**
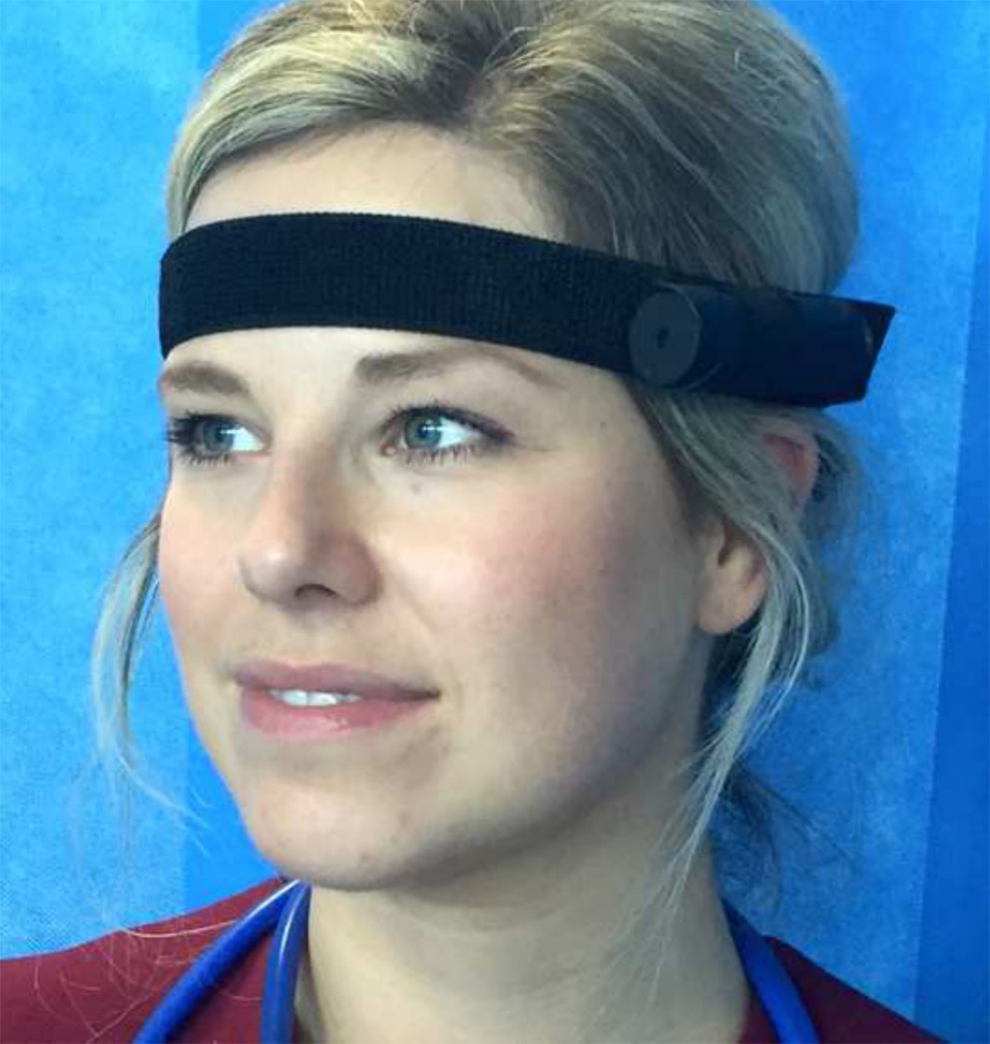
Research participant wearing a digital video camera

**Fig. 2:**
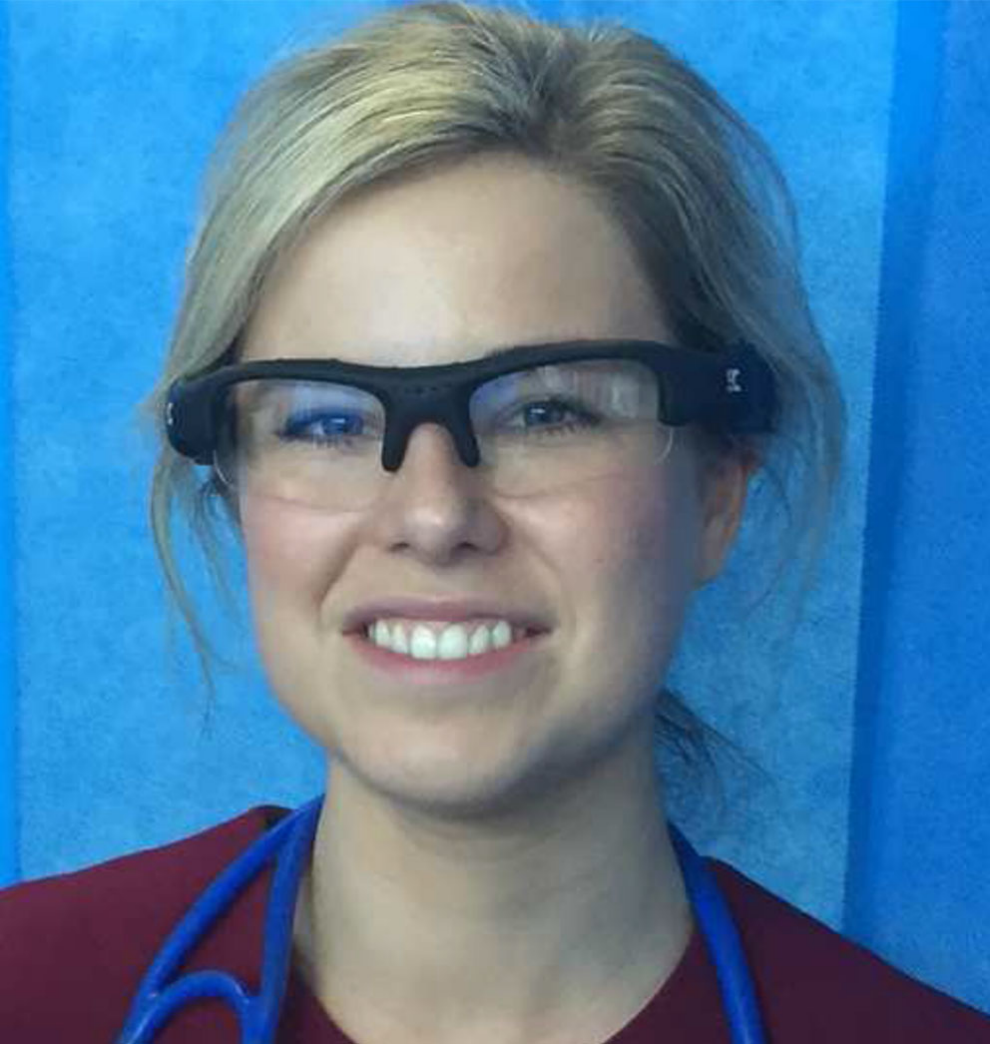
Research participant wearing a pair of digital video glasses

The traditional face-to-face interview is a fundamental research tool in qualitative research. Whilst this method of data collection can provide many insights into the subject’s experiences, it can often fall short of providing a complete picture [1]. Point of view (PoV) interviewing is an elicitation technique used in the social sciences as a means of enriching data obtained from research interviews. Recording a research subject’s first person viewings, either by attaching a digital video camera (Fig. 1) or wearing digital video glasses (Fig. 2), can afford deeper insights into their experiences. PoV filming can promote making *visible* the *unverbalizable* and does not depend as much on memory or recall as the traditional interview. It is less subject to the vagaries of post-hoc rationalization. Furthermore the interviewer is afforded the opportunity to observe and empathize with what the subject was actually seeing during a particular activity that is being researched. The use of this relatively easy technique and inexpensive technology is gaining interest in health profession educational research and pedagogy. In this interview, Dr Gerry Gormley (a medical education researcher) talks to Dr Jonathan Skinner (an anthropologist with an interest in PoV filming), exploring some of the many crossover implications with PoV interviewing for medical education, such as gaining a deeper understanding of what students experience in a dynamic simulation-based learning environment.

### Can you explain to me the concept of first person perspective digital recording/ethnography?

First person PoV filming continues the long tradition of filming in disciplines such as anthropology that began with filming fieldwork in the early twentieth century and extended to giving respondents cameras to film their social worlds, decolonizing the researcher’s gaze. This particular twenty-first century form of filming development devolves the camera to the respondent, capturing an approximation of what they see or the direction they are looking in through a digital camera fixed to the head, body or as digital video glasses serving as a third eye. It is a practice linked to the development and proliferation of digital media. As Pink points out, the camera records a trace through the participant’s environment; it enables one to empathize with the intensity of the entanglement with place committed, but it is not the exact bird’s eye view [1]. It is a version of the vision. This can be naturalistic such as fieldwork in a real clinical environment (for example how a patient experiences healthcare professionals during an acute stoke treatment pathway) or in a simulation-based learning activity (for example how a senior medical student manages in a ward-based simulation learning exercise that aims to develop their communication and human factor skills). The clinical scenarios are our field site, and the digital recordings are a visual text of our study: “the native point of view”.

### How long have first person perspective recordings, as a type of digital ethnography, been used in anthropology?

First person perspective recordings are a recent development in digital ethnography. The *reflexive turn* in contemporary ethnography has been matched by a *reflective turn* to the visual with the boom in personal media devices such as smart phone cameras and the reduction in pricing and size of digital cameras. Rather than interview participants and rely upon their recall and desire to re-present themselves, Georgiana Gore et al., a team of anthropologists in France who span anthropology, dance and sports science with their work on decision-making during social dramas (in Victor Turner’s sense of the word), used cameras to ‘elicit the tacit’ [2]. Theirs is a cognitive anthropology examining the underpinnings of bodily practices as they unfold. They used cameras to record activities such as refereeing a rugby match, and teaching a cookery class and a yoga class. The key participants are then confronted with the footage as a technique of elicitation – explicitation in their eyes – a verbalization of implicit knowledge. It is a data-mining of subjectivity and, with the interview aspect, there is a targeting of the narrations of the inner consciousness.

### How can you see this method being used in health profession education research? Do you have any examples that you can share with us?

Though first person PoV interview elicitation is becoming well established in the social science evidence base, there is still a gap in the health profession education literature. The PoV elicitation method has a range of possibilities and applications to it, not least in health profession educational research. It gives a more dramatic dimension to simulation-based learning activities, communications skills training environments, procedural skills development activities and team-based learning activities.

**Fig. 3:**
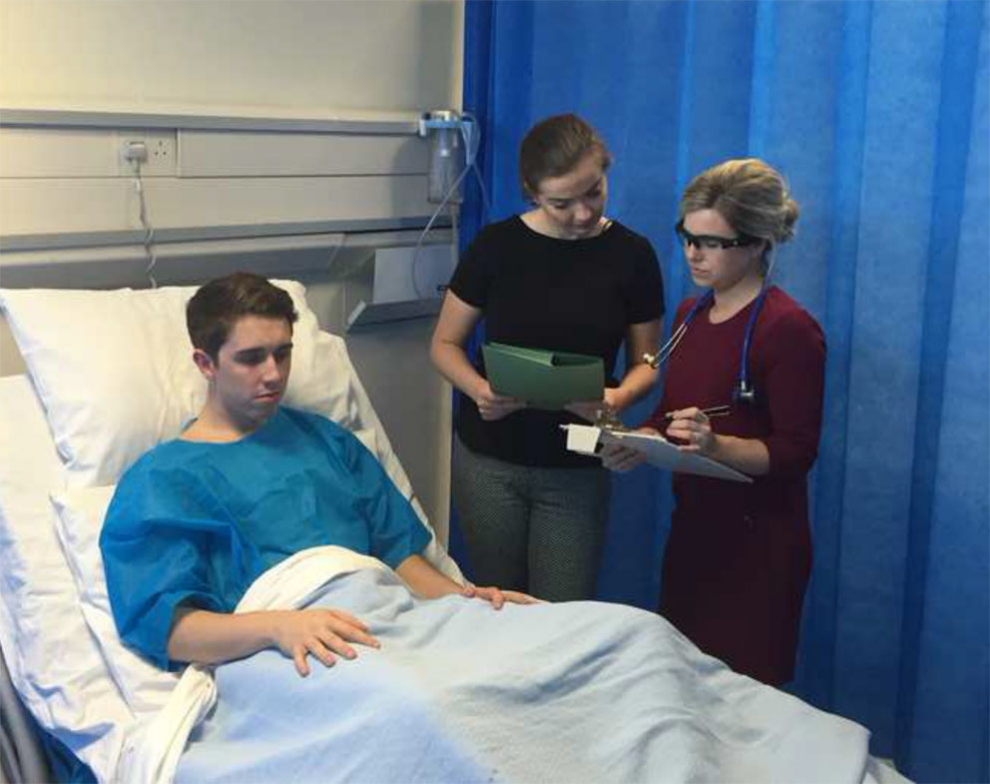
Research participant involved in a ward-based simulation research project whilst wearing digital video glasses

To give examples, we have had two iterations so far with students in Queen’s University Belfast and at the University of Roehampton. In the first, medical and nursing students took part in a ward-based simulation exercise that focused on human factors skills and team-based working. They had to achieve a range of certain clinical tasks but also had to cope with non-technical demands such as distraction and interruptions (Fig. 3).

**Fig. 4:**
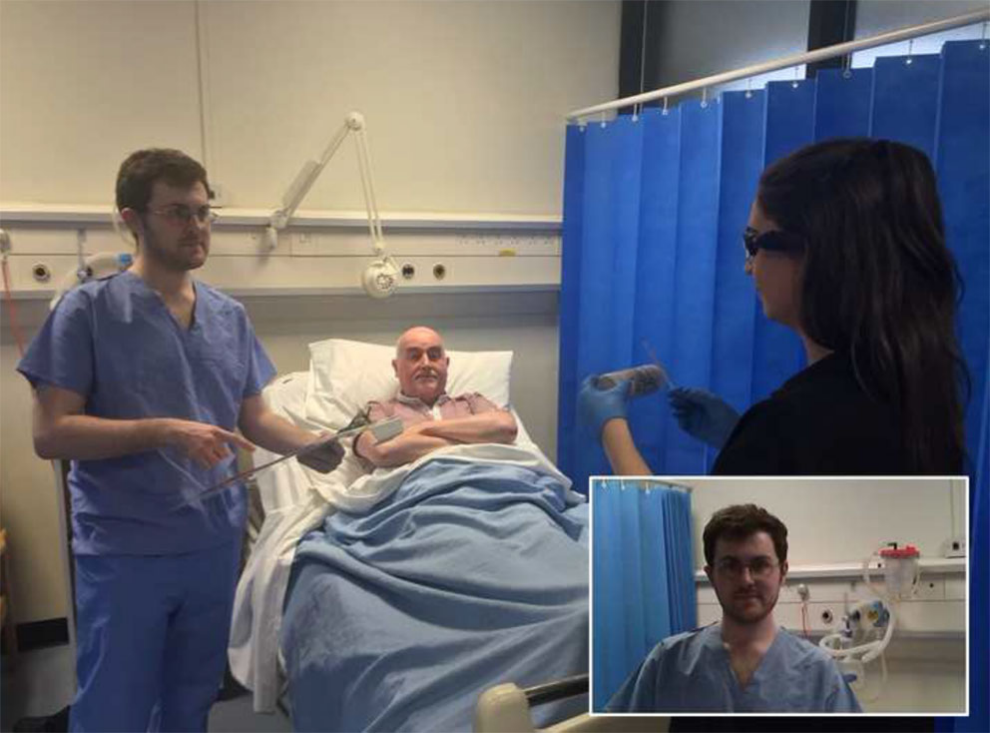
Medical student taking part in a ward-based simulation exercise (PoV image in bottom right corner of this image). Whilst the student is interpreting a urinalysis reagent stick, she is being interrupted not only by the patient but also by a nurse with a critical piece of clinical information about another patient

They saw that their field of vision was focused to the exclusion of peripheral events going on around them. However, these ‘peripheral events’ (e. g. in another part of the ward a patient’s clinical condition was deteriorating; or distraction by relatives and colleagues) were often not immediately picked up by the student but had a significant impact on the student’s actions (Fig. 4).

The second set of simulation-based learning activity, with University of Roehampton students and junior doctors from the University Hospital College London, followed decision-making processes in an ‘acute stroke treatment pathway’ simulation-based learning activity. These clinical scenarios are dynamic as patients are ‘transferred’ between the Emergency Department, Imaging Department and Resuscitation Room. The project examined empathy in the context of patient/doctor relations, as well as decision-making points for the doctors. Participants in these examples re-evaluated their performance after seeing themselves, in most cases seeing that they handled their performance in the simulation activity with more confidence and authority, skill and rapport than they had previously thought. In this situation, static wall-mounted cameras would not have captured the dynamic flow of the activity. Furthermore, a hand-held camera, by a third person, would have unnecessarily interfered with the emergent activity and not captured much of the close-up interactions between the student and the patient.

### Could such a technique and technology have any practical pedagogical uses?

It already has: elicitation techniques and experiential learning are ‘deep’ learning engagements, immersive and involving for the students. They are a way of teaching through practice for the instructor. We found the PoV footage can be used to assist, correct, reinforce, explain and praise practice. Digital video footage can be examined and re-examined, coded and ‘digested’. In short, real-life learning practice, and problem-based exercises and assignments, push the parameters of pedagogy: they capture the imaginations of the students and motivate them to engage over, above and beyond the norm. Feedback from the students showed not just effective and engaged, immersive teaching and learning, but also that the students were ‘valued’, ‘respected’ and ‘trusted’ as colleagues working together and not just as individuals chasing good grades. Altogether, it comes highly recommended by the students themselves.

### What does PoV filming bring to the traditional research interview?

The traditional interview is very useful and good at providing insights, and in complementing other research techniques such as participant observation. But it is far from ideal. It can miss a lot and is influenced by the media-driven mediated ‘interview society’ we now live in [3]. The quality of the interview depends on the ability of the interviewer and also the skills and guile of the interviewee, such as a politician, for example, someone media trained in the arts of turning a question or creating a word bridge from one part of the question to another to get their message across. The PoV interview is better at making *visible* the *unverbalizable* and, because it does not rely so much on memory, it is less subject to post-hoc rationalization. The elicitation interviews we are doing add to the triangulation of the subject. They are insights into the sense-making process of the participant as lived through and as they voice aloud their actions with the researcher. It allows the *interviewer* to see, with his/her own eyes, an approximation of what participants were actually experiencing during their activity. This can allow the *interviewer *to gain a greater connection with the *participant’s* experiences and so promotes a deeper shared understanding that is lacking in static walled-mounted cameras that might lose the front view of participants involved in a dynamic and fluid activity or lead to participants falling out of frame entirely (for example in a ward-based simulation-based learning exercise, participants may walk out of view from the static camera or have their back turned to the camera, and their actions will not be captured by the camera).

### What do you see as some of the challenges in the traditional interview in qualitative research and what advantages PoV interviewing can bring?

Interviewing, in the traditional sense, relies very much on the relationship between the interviewer and interviewee, as well as the interviewer’s knowledge and involvement in the subject area of the interviewee. You need to be able to check, respond, probe with your questions. Interviewers need an ability to read the interview context: body language, word stress, the ‘presence’ of the interviewee – are they there and engaged with you? Using a PoV camera gives one the ability to download and review the interview; to see it from different perspectives, even if both interviewer and interviewee are filming. It can help with skills building, allowing the student to practice on their rapport, their manner and delivery of questions, their proximity and presence with the interviewer that ranges from body language to engagedness; the perspicacity of their ‘conversation with a purpose’, as Kvale refers to this meaningful ‘*inter-change of views’*, quite literately an ‘*inter-view’* [4].

### What are the practical issues with using PoV?

The practical issues surround the price and type of technology one can afford. We started with basic ‘bullet’ videocams (Fig. 1) that were attached to the subject’s head. More recently, we have used Pivothead digital video glasses that are high definition, have excellent sound quality and are increasingly affordable (Fig. 2). The advantage of the digital glasses is that they feel like a pair of glasses so that the wearer is more comfortable with them on: they can get more immersed in the simulation. Also, the filming is from between the eyes rather than angled from the side of the forehead. When selecting the right equipment, we had to bear in mind the quality of image capture, its definition and the speed of filming and processing – how easy and fast it was to download and play during the subsequent interviewing.

In terms of ethics and governance, all research should have protocols put in place to protect research subjects, and no less for working with PoV interviewing. In terms of ethics, consent comes from making the people you are working with comfortable and feeling safe – knowing that they can trust you and that there are guidelines that you are working to for their safety. Briefings have been invaluable in achieving this, bringing the team together, showing how the technology works and explaining what you are trying to do with it and where and how you are using the results. The filming of simulation-based learning activities is complicated in that one has to be careful not to inadvertently capture footage of individuals unaware of the filming. All those in the simulation setting need to be aware of the filming. Continuous consent, an opt out, and the veto are ways to devolve controls away from the researcher. The nature of the research data also means that it needs to be kept very carefully so as to avoid corruption or leakage/exposure. Outside of a simulation learning environment context, in an actual clinical environment, the challenge of unpredictability is compounded. In that situation, the patient’s safety, security, trust and comfort need to be preserved at all costs.

### Are participants, or those they interact with, influenced by wearing the video head cameras?

Indeed. The camera wearers are ‘wary’ of the cameras, particularly when they first start to use them. In practical terms, we would place the cameras on the subjects before the activity to allow sensitization and adaption to the cameras. After switching the camera on, we would allow the camera to record continuously throughout the activity. Subjects soon find the immersion of the activity or simulation-based learning activity taking over and there are moments when we see that they have forgotten that they are wearing them, especially when they try to walk off still wearing the digital glasses! It’s the same with those they interact with. We make sure that all those involved in a simulation-based learning activity are briefed as to how the cameras work and that they have given their informed consent. There is a natural concern that no one is going to end up looking silly or end up on social media websites. These issues can be resolved by working to a continuous consent process with subjects: in several cases, participants deferred judgement until after the filming, or they made conditions that they were happy for academic publication but not for general public consumption of images or footage.
